# Primary Esophageal Extranasal NK/T Cell Lymphoma With Biphasic Morphology: A Case Report and Literature Review

**DOI:** 10.1097/MD.0000000000001151

**Published:** 2015-07-17

**Authors:** Zi-Yin Ye, Qing-Hua Cao, Fang Liu, Xiao-Fang Lu, Shu-Rong Li, Chang-Zhao Li, Shao-Hong Chen

**Affiliations:** From the Department of Pathology, The First Affiliated Hospital of Sun Yat-sen University (Z-YY, Q-HC, X-FL); Department of Oncology, Nanfang Hospital of Southern Medical University (FL); Department of Radiology, The First Affiliated Hospital of Sun Yat-sen University, Guangzhou, China (S-RL); Department of Dermatology and Skin Diseases Research Center, University of Alabama at Birmingham, AL, USA (C-ZL) and Department of Pathology, Guangzhou First People's Hospital, Guangzhou, China (S-HC).

## Abstract

We report a case of esophageal extranasal NK/T cell lymphoma with biphasic morphologic features revealed by a deep large piecemeal biopsy.

A 40-year-old man present with pharyngalgia, dysphagia, recurrent fever, and 5-kg weight loss for 8 months. Endoscopy demonstrated progressing longitudinal ulcers and mucosal bridges along the esophagus. The first and second biopsies obtained superficial mucosa with scattered bland-looking small lymphocytes. A subsequent large piecemeal snare abscission for biopsy showed atypical lymphoid cells infiltrating into the deep lamina propria and muscularis mucosae, whereas the superficial lamina propria was highly edematous with scant small lymphocytes. Immunohistochemical studies confirmed that both underlying atypical cells and superficial small lymphocytes were neoplastic, sharing an identical immunophenotype: positive for CD2, CD3, CD43, CD8, CD56, TIA-1 and granzyme B. Epstein-Barr virus–encoded small RNAs were found in both cells. The histologic findings were diagnostic of primary esophageal extranasal NK/T cell lymphoma. However, the patient developed bone marrow depression during chemotherapy and died of massive cerebral hemorrhage after the first cycle of chemotherapy.

Primary esophageal extranodal NK/T cell lymphoma nasal type is extremely rare. We show the biphasic morphology of this disease, which highlights the importance of deep biopsy for accurate diagnosis.

## INTRODUCTION

Primary esophageal lymphoma is rare, accounting for <1% cases of gastrointestinal lymphomas. B cell lymphomas are the most common histological subtype.^1,2^ Our review of the medical literature revealed only 3 cases of primary esophageal extranasal NK/T cell lymphoma published so far.^3,4^ Extranodal NK/T-cell lymphoma is characterized by diffuse infiltration of atypical lymphoid cells, angiocentric and angiodestructive growth pattern, coagulative necrosis, and admixed apoptotic bodies.^5^ Here, we report a case of primary esophageal extranasal NK/T cell lymphoma showing biphasic morphology and highlight the importance of deep biopsy for accurate diagnosis of tumor arising from the deep layer of esophageal wall.

## CASE REPORT

### Clinical Findings

A 40-year-old man was admitted for gradually aggravated pharyngalgia and dysphagia for 8 months in addition to recurrent fever and 5-kg weight loss. Results of physical examination were unremarkable, with no palpable lymphadenopathy, ascites, or organomegaly. A complete blood count showed a white blood cell count of 6.67 × 10^9^ cells/L, a red blood cell count of 5.47 × 10^12^ cells/L, a hemoglobin level of 100 g/L, and a platelet level of 216 × 10^9^ cells/L. The serum lactate dehydrogenase (LDH) level was 226 U/L (normal range 114–240 U/L), the total serum protein level was 69 g/L (normal range 64–87 g/L), and the serum albumin level was 35 g/L (normal range 35–50 g/L). Other laboratory values were within normal limits. Endoscopy demonstrated multiple esophageal ulcers, well-demarcated, with the largest measuring approximately 2.0 × 0.6 cm in cross-section. A biopsy was taken for pathologic examination and a diagnosis of chronic esophagitis was made. However, this patient showed no response to antibiotics administration.

**FIGURE 1:**
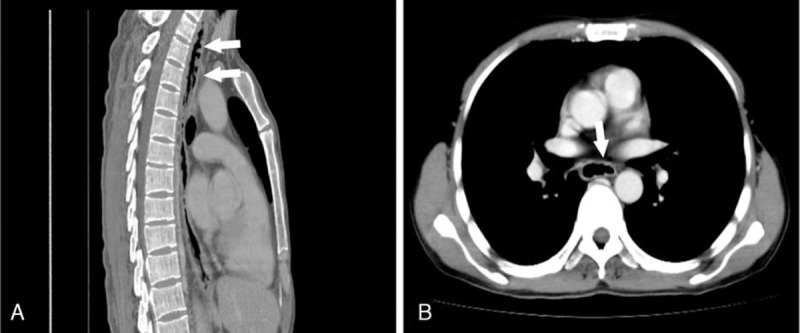
Computed tomography (CT) scan of an esophageal lesion. CT showed the esophagus wall was rigid and demonstrated rugosity (arrows) in the sagittal view (A) and transverse section (B).

Three months later, he was admitted again for recurrent pharyngalgia, sharpened retrosternal pain, and continuous fever. Laboratory tests, including blood counts and hepatic and renal function tests, remained stable. Chest computer tomography (CT) scan showed that the esophagus wall was rigid and incrassated. The inner wall was rough and uneven on the surface. After contrast administration, the CT scan showed multiple mucosal interruptions with enhancement occupying more than half of the esophageal wall (Figure 1). No enlarged lymph nodes were detected. CT scans of the head, neck, abdomen, and pelvis did not detect enlarged lymph nodes. The liver and spleen were of normal size and shape. Endoscopy revealed multiple deep ulcers along middle and distal portion of esophagus, with 3 deep longitudinal ulcers measuring 2 × 10 cm. A biopsy was taken and the pathologic diagnosis was chronic nonspecific esophagitis. No lesions appeared in the nasal cavities on nasal endoscopic examination.

**FIGURE 2:**
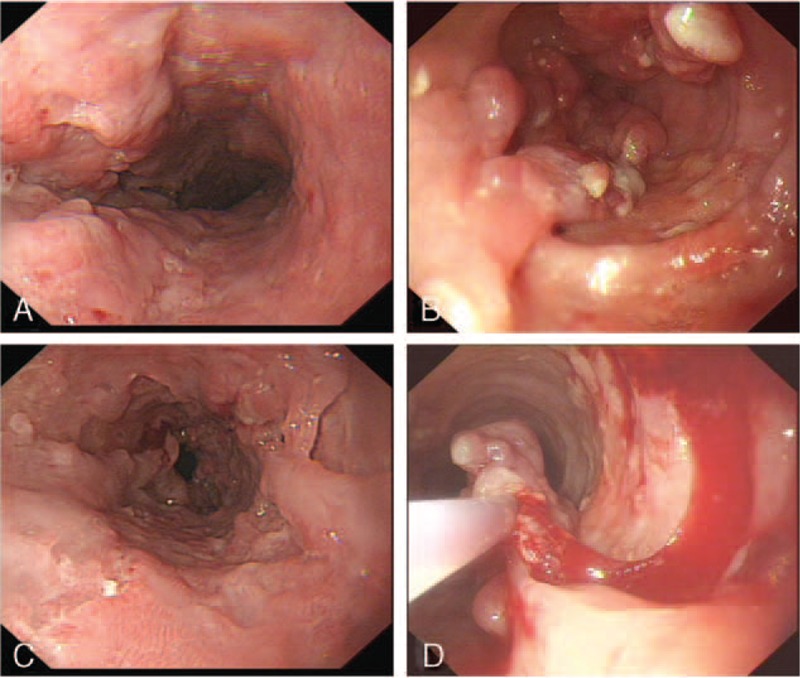
Endoscopy examination of esophageal lesion. Endoscopy demonstrated longitudinal ulcer (A), polypoid lesions (B), and longitudinal mucosal bridges (C). A large piecemeal snare abscission was performed to obtain sufficient tissue for diagnosis (D).

The patient had severe worsening of his original symptoms, and decreasing ability to swallow in the following 2 months. Of note, he had lost 7 kg since the first admission. Blood test showed his red blood cell count and hemoglobin level dropped to 3.50 × 10^12^ cells/L and 75 g/L, respectively. His LDH level was elevated to 370 U/L (normal range 114–240 U/L), his total serum protein level was decreased to 38.2 g/L (normal range 64–87 g/L), and his serum albumin level was 20.8 g/L (normal range 35–50 g/L). Repeated endoscopy showed mucosal erosion along the esophagus, approximately 17 to 40 cm from incisors. There were multiple polypoid lesions and longitudinal mucosal bridges with ulcers. A shallow longitudinal ulcer in the posterior wall was observed (Figure 2A–C). Following a large piecemeal snare abscission, a large portion of tissue was obtained for biopsy (Figure 2D). The final diagnosis was primary esophageal extranasal NK/T cell lymphoma. A bone marrow biopsy was subsequently performed and was negative for tumor involvement. In accordance with the Ann Arbor classification system, this case was classified as a stage IE disease.

One month after diagnosis, this patient was treated with chemotherapy using CHOP regimen (cyclophosphamide, doxorubicin, vincristine, and prednisone). However, he developed bone marrow depression with fever and interspersed cutaneous petechia on the 10th day of the first cycle of chemotherapy. Consistently, blood tests showed a WBC count of 0.5 × 10^9^ cells/L, a neutrophil count of 0.2 × 10^9^ cells/L, a hemoglobin level of 90 g/L, and a platelet level of 15 × 10^9^ cells/L. Three days later, he died of massive cerebral hemorrhage suddenly.

## MATERIALS AND METHODS

The specimen was fixed in a 10% neutral formalin solution and embedded in paraffin. Four-micromoles per liter sections were prepared for hematoxylin and eosin (H&E) staining or immunohistochemical (IHC) staining. An Envision 2-step assay was used for the IHC staining. Primary antibodies CD20, CD79a, CD2, CD3ε, CD5, CD4, CD8, CD43, CD56, TIA-1, Granzyme B, Ki-67, and horseradish peroxidase-conjugated secondary antibodies were obtained from DAKO Inc, Glostrup, Copenhagen, Denmark.

In situ hybridization (ISH) was performed to test for the presence of Epstein-Barr virus–encoded small RNA (EBER) in formalin-fixed, paraffin-embedded sections using a hybridization kit (DAKO).

For cytogenetic analysis, the paraffin tissue DNA was prepared with a tissue DNA extraction and purification kit (Dneasy TM Tissue Kit, Qiagene, CA). T-cell receptor rearrangement studies were performed.

This study was approved by the Human Ethics Committee of The First Affiliated Hospital, Sun Yat-sen University. Written informed consent was obtained from the patient's direct relative for publication of this Case Report and any accompanying images.

### Pathologic Findings

**FIGURE 3:**
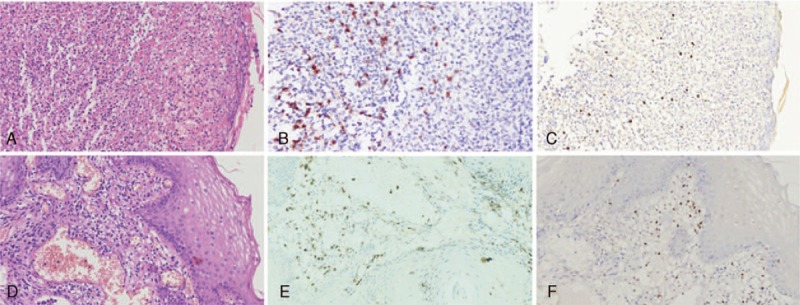
Pathological features of the first (A, B, C) and second (D, E, F) biopsies. The first biopsy obtained demonstrated ulceration with inflammatory exudate (A, H&E). Immmunohistochemical (IHC) staining (B) and in situ hybridization (ISH) for EBER (C) highlighted scattered positive lymphocytes. The second biopsy obtained fragments of squamous epithelium and superficial lamina propria with scattered small lymphocytes (D, H&E), which were positive for CD3 (E) and EBER by ISH (F).

The first biopsy obtained showed only ulceration with an inflammatory exudate and fragments of squamous epithelium (Figure 3A). Pathologic diagnosis was rendered as chronic nonspecific esophagitis without performance of IHC.

The second biopsy obtained fragments of squamous epithelium and superficial lamina propria with ulceration. Scattered small lymphocytes without atypical features were found in lamina propria (Figure 3D). IHC staining was not performed. The working pathologic diagnosis remained as chronic nonspecific esophagitis.

**FIGURE 4:**
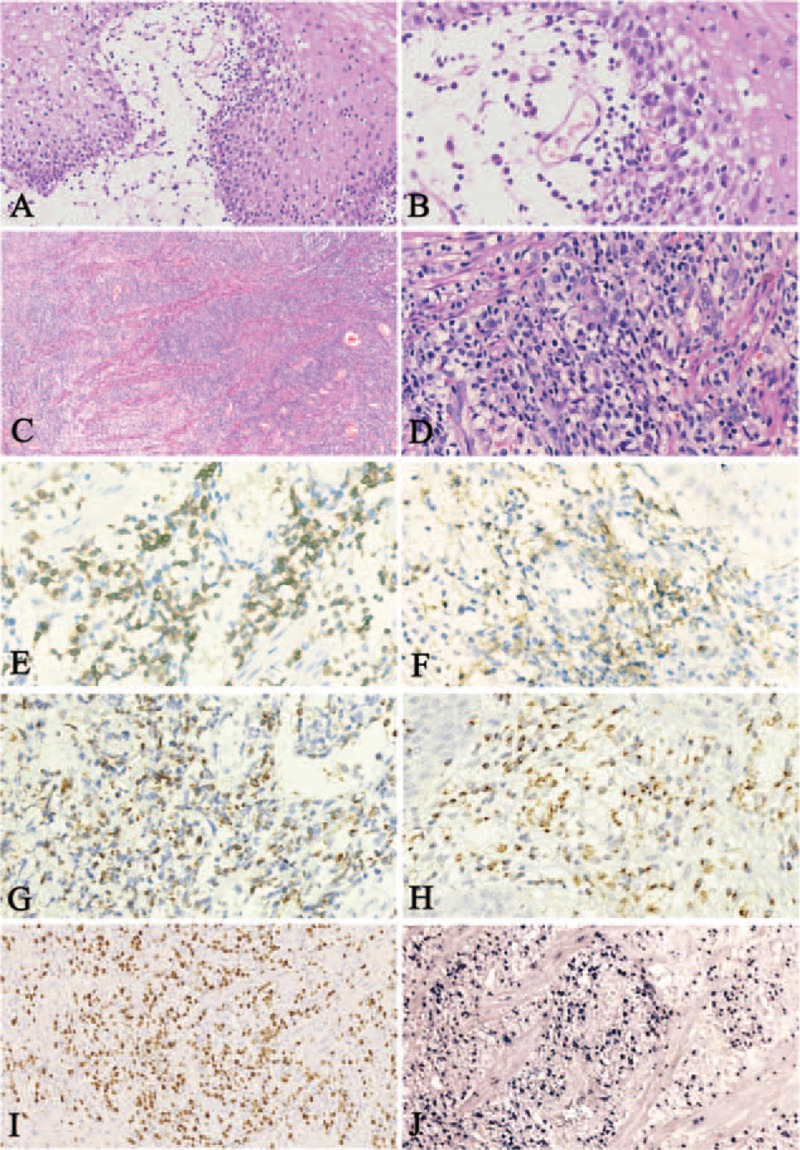
Pathological features of the third biopsy. The superficial lamina propria was highly edematous with scant small lymphoid cells (A and B, H&E). Atypical lymphoid cells infiltrated the muscularis mucosae (C and D, H&E). Tumor cells were positive for CD3 (E), CD56 (F), Granzyme B (G), TIA-1 (H), Ki67 (I), and Epstein-Barr virus–encoded small RNAs (J).

The last biopsy was taken with a large piecemeal snare abscission. Therefore, a large fragment of tissue was obtained. Histopathologic examination showed a biphasic population of lymphocytes in the superficial and deep lamina propria. The superficial lamina propria was highly edematous with scant small lymphocytes. These lymphocytes showed round condensed nuclei and rare mitotic figures were noted, practically identical to the lymphoid cells seen in the previous biopsies (Figure 4A, B). Focal ulceration was observed. However, numerous atypical lymphoid cells diffusely infiltrated into the deep lamina propria and muscularis mucosae, dispersing muscular bundles. The cells were small to medium-sized, with round hyperchromatic nuclei with inconspicuous nucleoli (Figure 4C, D). Neither an angiocentric infiltration pattern and nor an angiodestructive growth pattern was identified in the biopsy. Coagulative necrosis and admixed apoptotic bodies were not seen either. Immunohistochemical staining indicated tumor cells were positive for CD2, CD3 (Figure 4E), CD43, CD8, CD56 (Figure 4F), granzyme B (Figure 4G), and TIA-1(Figure 4H). A small portion of cells were positive for CD5. The proliferation index was approximately 60% as assessed by Ki-67 staining (Figure 4I). ISH for EBER showed strong positive signals in tumor cells (Figure 4J). The superficial small lymphocytes without atypical morphology were confirmed to be neoplastic because they also showed positive staining for EBER and shared the same immunophenotype with the atypical lymphoid cells beneath. No clonal rearrangement of T cell receptor genes was found by polymerase chain reaction heteroduplex analysis and polyacrylamide gel electrophoresis.

After the diagnosis of primary esophageal extranasal NK/T cell lymphoma based on the third biopsy, supplemental immunohistochemical staining and EBER ISH have been performed on tissues of the first 2 biopsies. Scattered lymphocytes were positive for CD2, CD3 (Figure 1B, E), TIA1, and EBER ISH (Figure 1C, F), suggesting the bland appearing lymphocytes, which were originally interpreted as benign were in fact neoplastic.

## DISCUSSION

Primary esophageal lymphoma is very rare, with only 40 cases reported in the English literature. The diagnosis of primary esophageal lymphoma should meet the following 5 criteria: no concomitant palpable superficial lymph nodes, no mediastinal lymphadenopathy, normal white blood cell count, no hepatic or splenic involvement, and the presence of an esophageal lesion.^6^ The most common subtypes of primary esophageal lymphomas reported are extranodal marginal zone lymphoma of mucosa-associated lymphoid tissue (MALT lymphoma)^1,7–11^ and diffuse large B-cell lymphoma.^12–14^ Aside from B cell lymphomas, there were also 10 cases of T or NK-cell lymphoma^15–18^ and 3 cases of Hodgkin lymphoma^19,20^ reported. Most primary esophageal lymphomas arose in patients older than 50 years. The present symptoms, such as dysphagia epigastric pain and weight loss, were nonspecific. Endoscopic findings were variable and included submucosal tumor infiltration, polypoid growth, and ulceration. MALT lymphomas commonly presented as submucosal tumors with stage I disease. The most common treatment was endoscopic mucosal resection or endoscopic submucosal dissection with or without chemotherapy and radiotherapy. Most patients showed no evidence of recurrence on follow-up (1–3 years). Aggressive lymphomas, such as T-cell lymphomas and diffuse large B-cell lymphomas, commonly presented as tumor masses with obstruction and ulceration on endoscopy. Treatment included chemotherapy, with or without surgery, and radiotherapy. Prognosis is variable and advanced stage disease shows a poor outcome.

Primary esophageal NK/T cell lymphoma is extremely rare, with only 3 cases reported previously (Table 1).^3,4^ The present case is the fourth primary esophageal NK/T cell lymphoma reported so far. The age range of the reported cases was 40 to 54 years. Endoscopy showed ulceration with or without masses. Initial treatments were chemotherapy with or without radiotherapy. All the patients died within 2 to 32 months. The previous reports simply focused on endoscopy appearance. In the present case, we highlighted the biphasic histopathologic features of the tumor with emphasis on the bland morphologic characteristic of tumor cells, which may be seen in superficial mucosal biopsies. We emphasized the necessity of obtaining deep mucosal tissue for biopsy to avoid a benign misclassification in these cases.

**TABLE 1:**
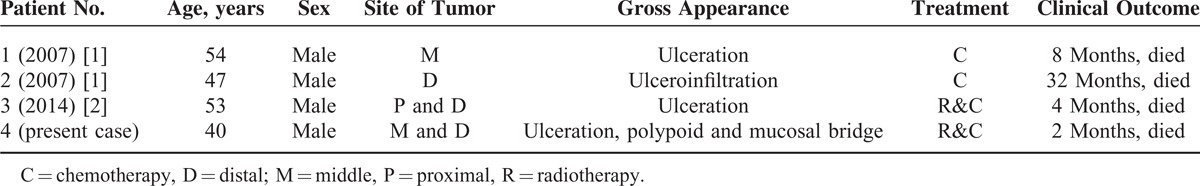
Clinical Profile of Reported Cases of Primary Esophageal Extranodal NK/T Cell Lymphoma, Nasal Type

Extranodal NK/T-cell lymphoma is much more prevalent in the Asian population as compared with the overall population.^21^ Nasal NK/T-cell lymphoma, referring to cases with primary tumor sites locating in the upper airway regions, including the nasal cavity, nasopharynx, paranasal sinuses, tonsils, hypopharynx, and larynx, has been reported to account for 60% to 90% of all extranodal NK/T-cell lymphomas.^22,22^ Extranasal NK/T-cell lymphoma is defined as the presence of primary tumor at all other sites in the absence of nasal disease, most often arising from the gastrointestinal tract, skin, lungs, or liver.^23^ Patients with extranasal NK/T-cell lymphoma had more adverse clinical features (eg, a higher stage, elevated LDH, more bulky disease, and poor performance status) and poorer survival rate compared with nasal cases, even in cases with apparently localized disease.^23–25^ However, there are no significant differences in the immunophenotypic or genotypic profiles between the nasal and extranasal cases.^26^

The most common feature of extranasal NK/T-cell lymphoma of gastrointestinal tract detected with endoscopy is an ulceroinfiltrative lesion. However, there are no pathognomonic signs to distinguish lymphoma from other malignancies or benign lesions, including Behçet disease or inflammatory bowel disease. Primary endoscopy detects malignant lesions in approximately 65% of gastrointestinal (GI) tract lymphomas later confirmed by biopsy.^26^ Endoscopic ultrasonography (EUS) has proven to be much more effective than general endoscopy in detecting GI lymphomas. This method is superior because of its high resolution and its ability to provide more accurate information as to the involvement of the deeper layers of the esophageal wall.^27^ Lymphomas arising from deep lamina propria or submucosa are beyond the reach of usual endoscopic biopsy, and may lead to misdiagnosis. EUS help reveal the exact location of tumor and guide precise biopsy. If EUS had been performed in this case prior to the first biopsy, it might have helped reveal that the main lesion present deep within the esophageal well and provided guidance to obtain a deeper and larger portion of tissue for biopsy. This may have avoided the initial pathologic misdiagnosis and the necessity for subsequent multiple biopsies. In addition, new endoscopic instruments, including magnifying endoscopy with narrow band images, autofluorescence imaging, and confocal laser endomicroscopy, will help increase diagnostic accuracy in the future.^28,29^

For those cases with masses arising from the superficial mucosa, general endoscopy is usually able to obtain diagnostic tissue with rare complications. However, for those tumors arising more deeply, general endoscopy biopsy may fail to get sufficient diagnostic tissue, which may lead to delayed treatment or misdiagnosis. In that case, a repeated biopsy for a deeper and larger portion of tissue is needed to reach an accurate diagnosis. Nevertheless, clinicians may be concerned with performing a deeper biopsy because deeper biopsies increase the risk for hemorrhage or perforation. As a result, a balance between obtaining sufficient tissue for diagnosis while minimizing the risk of iatrogenic complications should be achieved. Gastroenterologists should try to obtain a deeper and larger portion of tissue for accurate diagnosis while attempting to reduce the incidence of complications with close patient monitoring. For these purposes, EUS can aid during the procedure to avoid large blood vessels and damage to serosa during the biopsy procedure.

There are no significant differences in histopathologic features between nasal NK/T-cell lymphoma and extranasal NK/T-cell lymphoma. Medium to large-size atypical lymphoid cells diffusely infiltrate the tissue and usually demonstrate irregular hyperchromatic nuclei. Angiocentric and angiodestructive growth pattern, coagulative necrosis, and admixed apoptotic bodies are characteristic but not universal features.^5^ For example, the absence of an angiocentric or angiodestructive pattern is found in approximately 30% cases.^25^ In addition, the presence of angiocentric/angioinvasive pattern may be dependent on the size of biopsy specimen, with larger specimens more likely to show these findings. In the present case, biphasic morphology was seen in the esophageal biopsy. Scattered bland-appearing neoplastic cells resembling small lymphocytes infiltrated into superficial lamina propria with edema and were not recognizable as tumor cells by H&E staining alone. Immunohistochemical staining and EBER ISH were necessary to highlight the small tumor cells.^25^ Tumor cells with obviously atypical features were diffusely infiltrated into the deep lamina propria and muscularis mucosae, which were not seen on the initial biopsy. In these circumstances, superficial biopsies may lead to misdiagnosis as chronic inflammation. Diagnosis of esophageal extranasal NK/T-cell lymphoma will be difficult in some cases without a larger and deeper biopsy than that normally taken in routine endoscopy procedures.

The clinical differential diagnosis for esophageal extranasal NK/T-cell lymphoma includes Behçet disease, Crohn disease, and tuberculosis (TB). Behçet disease is characterized by the triple-symptom complex of recurrent oral aphthous ulcers, genital ulcers, and uveitis. It also involves visceral organs such as the gastrointestinal tract, lung, cardiovascular, and neurological systems. Esophageal involvement was found in 4.7% of cases.^30^ Endoscopy shows single or multiple punched-out ulcers with well-demarcated edges and relatively flat bottoms. Microscopic features include chronic active inflammation with ulceration.^31^ Although vasculitis had been listed as one of the diagnostic features, it is rarely found on biopsy. Crohn disease is a chronic relapsing and remitting inflammatory disease with multifocal involvement along the gastrointestinal tract. Esophageal involvement accounts for 6% of Crohn disease,^32^ characterized by aphthous ulcers, longitudinal ulcers, and strictures. Morphologic features include focal or patchy chronic inflammation and noncaseating granulomas. Esophageal TB is the most common secondary site of TB developed in the lymph node or lung, but it is a rare primary site for TB. Endoscopy of esophageal TB often shows ulceration or infiltrative growth in the lumen.^33^ However, microscopic features of TB include multiple granulomas, often characterized by their large-sized and coalescent architectural pattern. Caseation necrosis can be found in the center of large granulomas. Mycobacterial organisms can be identified with acid-fast stains.

The strategy for management of extranodal NK/T-cell lymphoma remains controversial. The current suggestion for management of extranodal NK/T-cell lymphoma is systemic chemotherapy and radiotherapy targeting the involved field if applicable.^34^ For patients with clinically localized nasal disease, radiotherapy has been regarded of paramount importance because about 70% of patients achieve complete remission after treatment.^35–37^ However, extranasal disease appears to be less amenable to conventional radiotherapy. Currently, chemotherapy is the primary treatment for patients with systemic disseminated disease. In this regard, CHOP is a very common regimen, but the prognosis is far from satisfactory.^38^ The 5-year overall survival was around 40% following CHOP regimen in combination with radiotherapy. Moreover, anthracycline-containing chemotherapy has a poor response in extranodal NK/T cell lymphoma. One study showed 71% of patients with extranodal NK/T cell lymphoma failed to achieve remission with CHOP therapy.^39^ Other nonanthracycline drugs (eg, methotrexate, L-asparaginase) have shown some promising effects on treating relapsed or refractory patients.^40,41^ Autologous or allogeneic stem cell therapy may provide a survival benefit for patients with extranasal or advance nasal diseases.^42,43^

## CONCLUSION

Primary esophageal extranasal NK/T cell lymphoma is extremely rare. We reported a case with unique biphasic morphology. The neoplastic cells present within the superficial mucosa had a bland morphological appearance and resembled small lymphocytes, which in these cases may easily be misdiagnosed as benign. A deeper biopsy is often necessary to reveal the underlying diagnostic morphologic features of this entity.
